# Supplementary figures and images for: Comparison of Gonadal Transcriptomes Uncovers Reproduction-Related Genes with Sexually Dimorphic Expression Patterns in Diodon hystrix
Source: Animals (Basel). 2021 Apr 7;11(4):1042. doi: 10.3390/ani11041042 (PMC8068034; doi:10.3390/ani11041042)

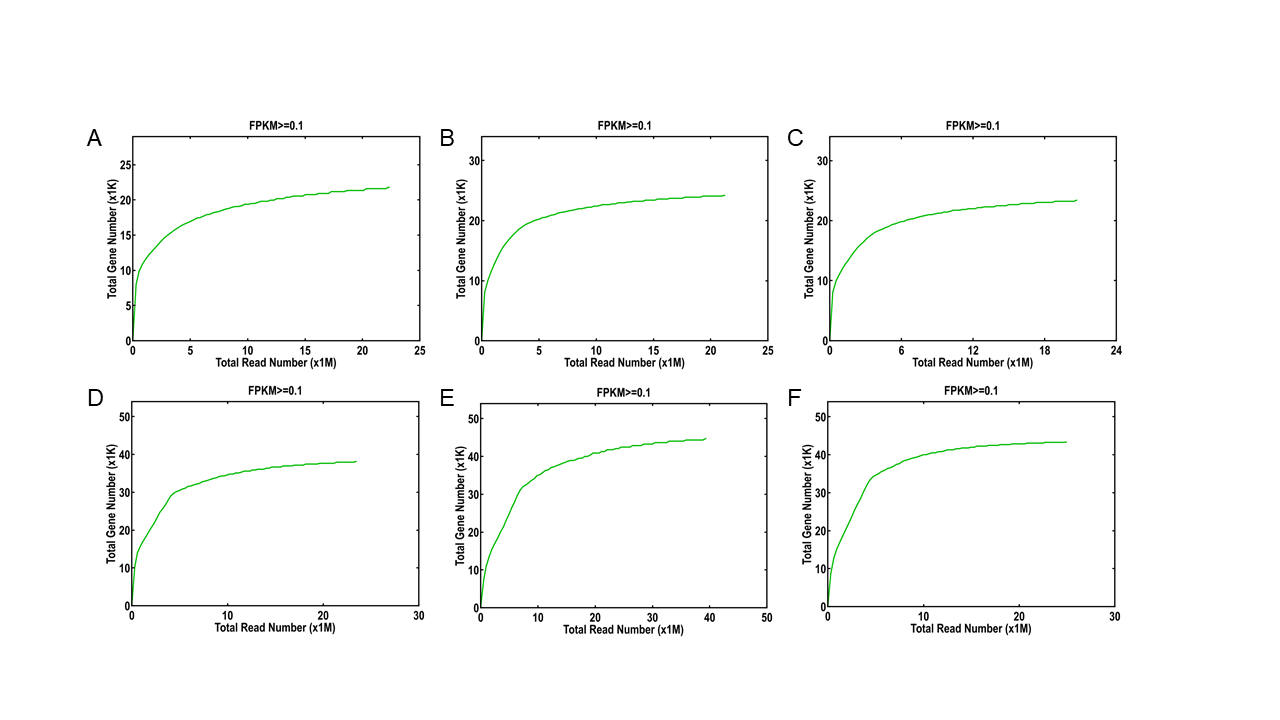

Supplement: Supplementary file 1 [file animals-11-01042-s001.zip › Supplementary Files/Figure S1.tif]

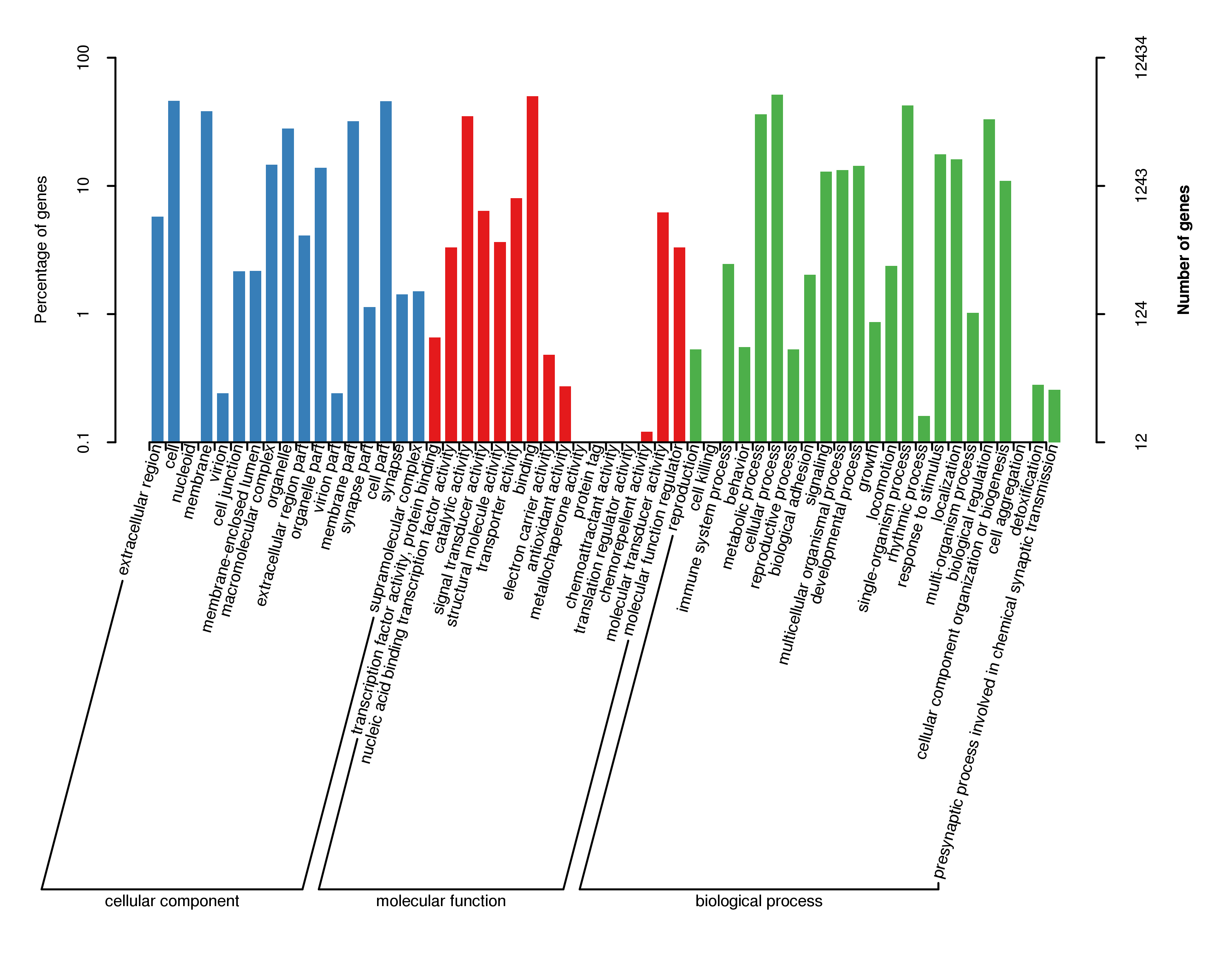

Supplement: Supplementary file 1 [file animals-11-01042-s001.zip › Supplementary Files/Figure S2.tif]

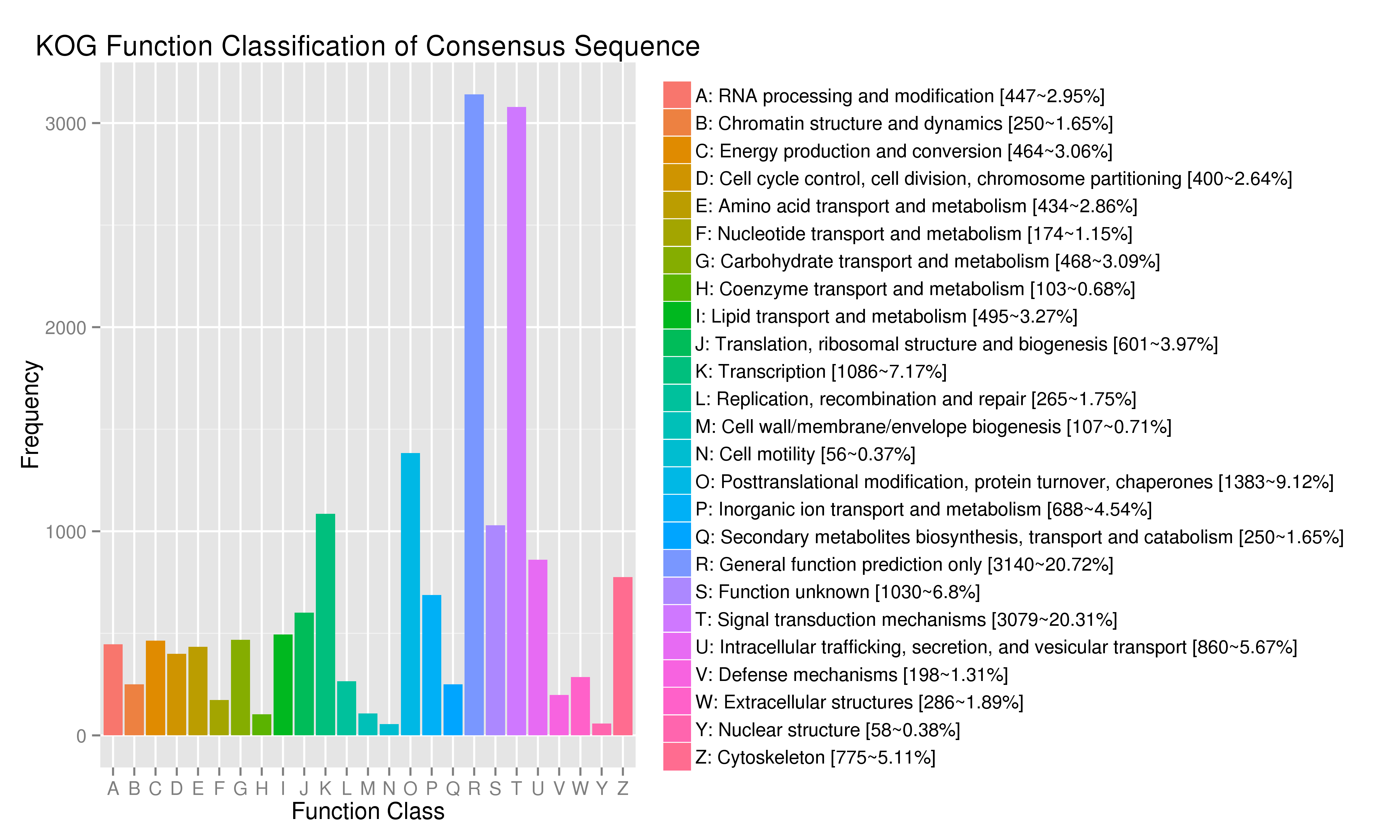

Supplement: Supplementary file 1 [file animals-11-01042-s001.zip › Supplementary Files/Figure S3.tif]

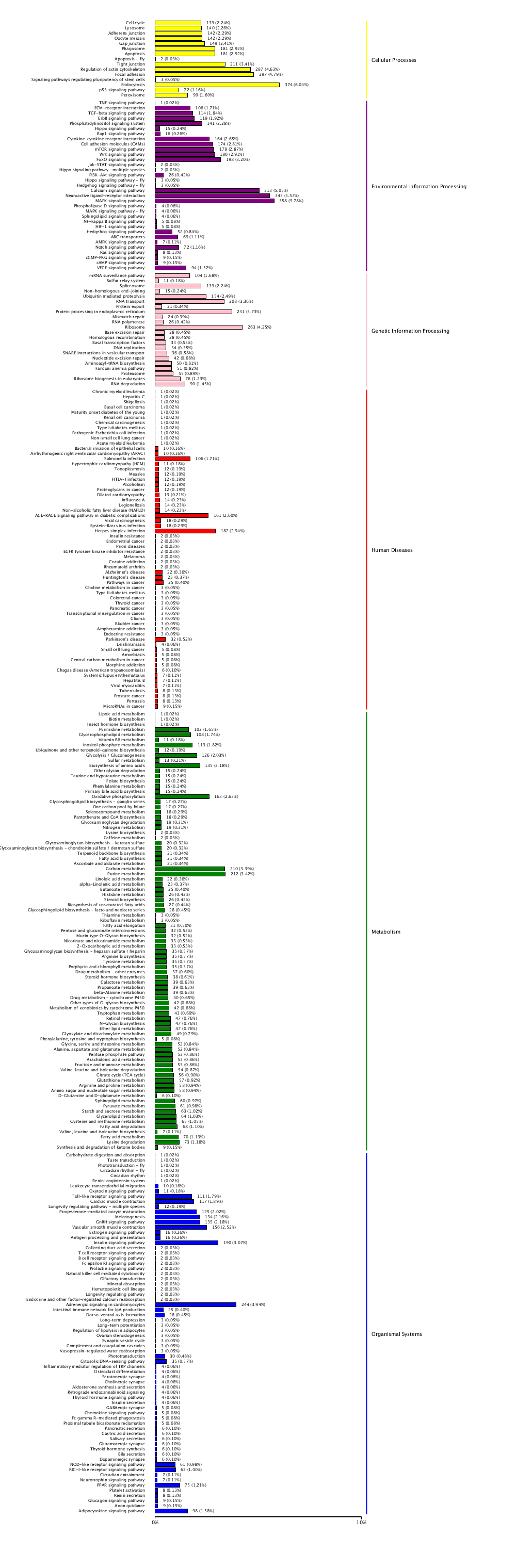

Supplement: Supplementary file 1 [file animals-11-01042-s001.zip › Supplementary Files/Figure S4.tif]

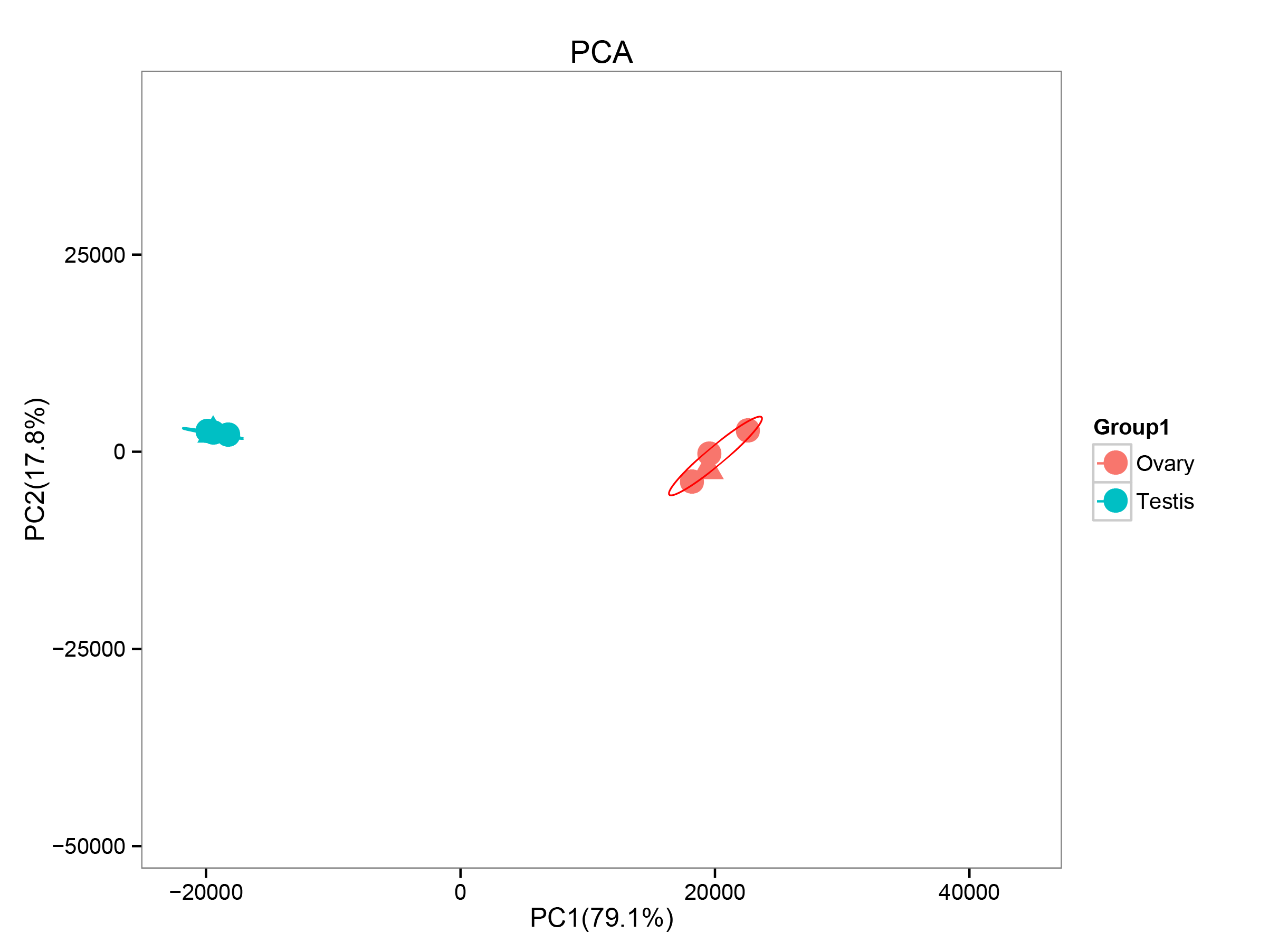

Supplement: Supplementary file 1 [file animals-11-01042-s001.zip › Supplementary Files/Figure S5.tif]
